# Evaluation of Prostate Cancer Screening Strategies in a Low-Resource, High-risk Population in the Bahamas

**DOI:** 10.1001/jamahealthforum.2022.1116

**Published:** 2022-05-20

**Authors:** Eveline A. M. Heijnsdijk, Roman Gulati, Jane M. Lange, Alex Tsodikov, Robin Roberts, Ruth Etzioni

**Affiliations:** 1 Department of Public Health, Erasmus MC, University Medical Center Rotterdam, Rotterdam, the Netherlands; 2 Division of Public Health Sciences, Fred Hutchinson Cancer Research Center, Seattle, Washington; 3 Knight Cancer Institute, School of Medicine, Oregon Health & Science University, Portland; 4 School of Public Health, University of Michigan, Ann Arbor; 5 University of The West Indies School of Clinical Medicine and Research, Nassau, The Bahamas

## Abstract

**Importance:**

The benefit of prostate-specific antigen screening may be greatest in high-risk populations, including men of African descent in the Caribbean. However, organized screening may not be sustainable in low- and middle-income countries.

**Objective:**

To evaluate the expected population outcomes and resource use of conservative prostate-specific antigen screening programs in the Bahamas.

**Design, Setting, and Participants:**

Prostate cancer incidence from GLOBOCAN and prostate-specific antigen screening data for 4300 men from the Bahamas were used to recalibrate 2 decision analytical models previously used to study prostate-specific antigen screening for Black men in the United States. Data on age and results obtained from prostate-specific antigen screening tests performed in Nassau from 2004 to 2018 and in Freeport from 2013 to 2018 were used. Data were analyzed from January 15, 2021, to March 23, 2022.

**Interventions:**

One or 2 screenings for men aged 45 to 60 years and conservative criteria for biopsy (prostate-specific antigen level >10 ng/mL) and curative treatment (Gleason score ≥8) were modeled. Categories of Gleason scores were 6 or lower, 7, and 8 or higher, with higher scores indicating higher risk of cancer progression and death.

**Main Outcomes and Measures:**

Projected numbers of tests and biopsies, prostate cancer (over)diagnoses, lives saved, and life-years gained owing to screening from 2022 to 2040.

**Results:**

In this decision analytical modeling study, screening histories from 4300 men (median age, 54 years; range, 13-101 years) tested between 2004 and 2018 at 2 sites in the Bahamas were used to inform the models. Screening once at 60 years of age was projected to involve 40 000 to 42 000 tests (range between models) and prevent 500 to 600 of 10 000 to 14 000 prostate cancer deaths. Screening at 50 and 60 years doubled the number of tests but increased lives saved by only 15% to 16%. Among onetime strategies, screening once at 60 years of age involved the fewest tests per life saved (74-84 tests) and curative treatments per life saved (1.2-2.8 treatments).

**Conclusions and Relevance:**

The findings of this decision analytical modeling study of prostate cancer screening in the Bahamas suggest that limited screening offered modest benefits that varied with screening ages and number of tests. The results can be combined with data on capacity constraints and evaluated relative to competing national public health priorities.

## Key Points

**Question:**

What are the expected population outcomes and associated medical resource requirements of conservative prostate-specific antigen screening programs in the Bahamas?

**Findings:**

In this study of 4300 men screened for prostate cancer, 2 decision analytical models projected modest effects of undergoing 1 or 2 prostate-specific antigen screening tests on prostate cancer incidence and mortality rates. These programs are expected to have more favorable harm-benefit ratios than in high-income countries.

**Meaning:**

Although the population outcomes of conservative prostate-specific antigen screening programs in the Bahamas are expected to be limited, the programs are expected to be more efficient than in high-income countries.

## Teaser

This decision analytical model of prostate cancer screening programs in the Bahamas evaluates the projected benefit of limited screening programs and the implied resources required for their sustainable implementation.

## Introduction

Managing prostate cancer is a global health challenge. Mortality rates are highest among men of African descent in the Caribbean (27.9 per 100 000 men), followed by middle Africa (24.8 per 100 000 men) and southern Africa (22.0 per 100 000 men).^1,2^ Prostate cancer among Black men in low- and middle-income countries often presents in an advanced stage, when treatment options are limited. Although prostate cancer screening trials have produced apparently divergent results concerning benefit, there is now a general consensus that screening confers a clinically significant reduction in prostate cancer mortality.^3,4^ Prostate cancer screening in high-risk populations has the potential to be highly beneficial^5,6^; however, low- and middle-income countries may not have the resources needed for screening and follow-up care.

The appropriate allocation of scarce health care resources in low- and middle-income countries goes beyond available resources. A cancer screening program should be not only feasible but also effective, equitable, cost-effective, and subject to quality assurance.^7,8^ Successful implementation may require adequate infrastructure not only for the program itself but also for improving awareness, educational initiatives, and administration of a cancer registry to track performance. A pragmatic first step is to assess the resources needed for potential screening protocols, given the number of tests, testing ages, and criteria for biopsy and treatment.^9^

In the Bahamas, where more than 85% of the population has African ancestry, the government provides 70% of health care services at a cost of $1063 per person, or 4.6% of per capita gross domestic product.^10^ Public health investments have achieved nearly universal vaccination for common childhood diseases, an infant mortality rate below 20 per 1000 live births, and a life expectancy of 75 years.^11^ However, prostate cancer remains the third leading cause of death.

Starting in 2004, the Cancer Society of the Bahamas introduced a community-based awareness campaign and free annual prostate-specific antigen (PSA) and digital rectal examination (DRE) tests on the 2 most populated islands of the Bahamian archipelago (New Providence [population 200 000] and Grand Bahama [population 52 000]), adhering to American Urological Association guidelines for prostate cancer screening for men of African ancestry.^12^ All men with biopsy-proven prostate cancer were offered treatment options in accordance with National Comprehensive Cancer Network guidelines. However, in general, there is limited access to and availability of cancer care services. Advanced imaging and radiation therapy services are available in the private sector only and are accessed by the public on a fee-for-service basis.^10^ These privately owned services often require out-of-pocket payment because only 30% of the population has private health insurance coverage.

This study investigated ways to sustainably reduce prostate cancer mortality in the Bahamas in the presence of these resource limitations. Adapting previously developed decision analytical models of prostate cancer screening to the Bahamian population, we projected the outcomes of conservative screening strategies most likely to be feasible in this setting. We used the screening program data to inform and validate the models and projected benefits along with the implied resources needed for screening, diagnosis, and treatment.

## Methods

### Prostate Cancer Screening in the Bahamas

The Cancer Society of the Bahamas introduced community-based prostate cancer screening using PSA and DRE testing in 2004. Every September, the local chapter of the international Us TOO Prostate Cancer Support Group organizes an intensive public campaign (by newspapers, television, radio, church announcements, town meetings, flyers, and telephone calls to men who attended previously), and men aged 40 years or older are offered free PSA and DRE tests in Nassau, New Providence, and Freeport, Grand Bahama.

For this study using 2 decision analytical models, we used age and PSA results from screening tests performed in Nassau from 2004 to 2018 and in Freeport from 2013 to 2018. All men attending the screening clinic were informed of their results by telephone within 2 weeks. Men with normal DRE results and PSA levels were encouraged to continue annual screening. All men with an abnormal DRE result or PSA level higher than 4 ng/mL (>2 ng/mL for <50 years of age) were directed to attend a follow-up visit with a urologist. According to age, PSA kinetics, repeated DRE testing results, health status, and family history, men were advised to return for the next annual screening, take another PSA test after 1 to 3 months, or proceed to a transrectal ultrasonographically guided 12-core biopsy in cases in which the PSA level was higher than 10 ng/mL or the DRE result was suggestive of cancer. For this study, we also reviewed Gleason scores of 207 biopsies.

### Two Models of Prostate Cancer Natural History, Diagnosis, and Survival

We used 2 established decision analytical microsimulation models, the Erasmus Medical Center Microsimulation Screening Analysis (MISCAN) model and the Fred Hutchinson Cancer Research Center (FHCRC) model.^13,14,15^ Both models describe transitions between clinical prostate cancer states and from latent disease to clinical diagnosis.

In the MISCAN model, cancer progresses through stages (cT1, T2, and T3) and grades (Gleason scores ≤6, 7, and ≥8). Progression rates are not explicitly correlated with PSA levels. In the FHCRC model, cancer grade (Gleason scores ≤6, 7, and ≥8) is fixed at onset, with older men more likely to have higher-grade disease. Rates of progression from latent to clinical and from localized to metastatic disease are correlated with PSA levels, with PSA levels and growth rates estimated using serial testing data from men in the Prostate Cancer Prevention Trial.^16^ Both models were previously calibrated to Surveillance, Epidemiology, and End Results incidence of prostate cancer for Black men in the United States.^17^

In both models, baseline prostate cancer survival for untreated Black men was based on survival among men receiving a diagnosis in the Surveillance, Epidemiology, and End Results database from 1980 to 1986 (eMethods, eTables 2 and 3, and eFigures 6 and 7 in the Supplement). We used these years for baseline survival because they fell just before the widespread dissemination of prostate cancer screening in the United States. Among localized cases, we assumed only men with high-grade disease (Gleason score ≥8) would receive curative treatment and applied a treatment hazard ratio of 0.55 based on the Scandinavian Prostate Cancer Group 4 trial of radical prostatectomy vs watchful waiting.^18^ For the effect of screening on disease mortality, we used a lead-time–dependent cure rate, which models the likelihood of cure among screen-detected cases as increasing with the earliness of detection. The cure rate was previously calibrated to the European Randomized Study of Screening for Prostate Cancer (ERSPC).^4^

### Adapting the Models to the Bahamian Population

To adjust the models to the Bahamian setting, we simulated other-cause death with a lifetable of the Bahamas in 2016,^19^ extrapolated to 84 to 94 years of age using Holt-Winters exponential forecasting.^20,21,22^ We used the age distribution of 70 440 men aged 40 to 84 years residing in the Bahamas in 2020.^23^ We recalibrated the transition rates to clinical diagnosis (keeping other natural history parameters fixed) to match prostate cancer incidence in 2018 by 15-year age groups in the Bahamas reported by GLOBOCAN.^2^

To validate the models, we compared the projected prostate cancer mortality in 2018 with the observed mortality reported by GLOBOCAN. We also simulated single screening tests and compared projected proportions of men with PSA levels higher than 10 ng/mL by age with empirical results from the 4300 first screening tests performed in Nassau and Freeport. Finally, we compared projected proportions of cancers with Gleason scores lower than or equal to 7 with the empirical data.

### Screening Strategies

Taking into account the limited resources for testing, biopsy, and curative treatment, we considered strategies with 1 or 2 screening tests and conservative criteria for biopsy and curative treatment.^9^ We simulated strategies consisting of a single screening at 45, 50, 55, or 60 years of age and 2 screenings at 45 and 55 years or at 50 and 60 years, all starting in 2022. Because potential adherence for these strategies is unknown, we assumed 100% attendance to screening and to 12-core systematic biopsy with 80% sensitivity.^24^ We used a PSA level higher than 10 ng/mL as a threshold for biopsy in all strategies.

### Statistical Analysis

For each strategy, we projected the age-standardized prostate cancer incidence and mortality from 2020 to 2040, as well as the number of screening tests, biopsies, cancers detected (overall and owing to screening), curative treatments, and overdiagnoses for the Bahamian population aged 40 to 84 years from 2022 to 2040. An overdiagnosed case is one in which cancer was detected by screening a patient who, in the absence of screening, would never have received that diagnosis within his lifetime. We also projected lives saved and life-years gained because of screening, which were calculated by comparing modeled ages and causes of death with vs without screening; these outcomes were attributed to screening during this period even if the benefit manifested after 2040. Data were analyzed from January 15, 2021, to March 23, 2022.

### Sensitivity Analysis

The outcomes with the greatest uncertainty were lives saved and life-years gained, and these outcomes were determined by the survival benefit mechanism. The cure-rate benefit was informed by the ERSPC, which included few men of African descent. To explore sensitivity to the assumed cure-rate benefit, we also considered a stage-shift formulation. Under this formulation, men who would have received a diagnosis of metastatic disease without screening but whose cancer was detected early by screening had prostate cancer survival that corresponded to the earlier stage, grade, or both. To ensure that this benefit excluded lead time and applied only to non-overdiagnosed cases, this survival began at the end of their lead time (ie, at their counterfactual diagnosis without screening).^25,26^

This decision analytical modeling study followed the Consolidated Health Economic Evaluation Reporting Standards (CHEERS) guideline where applicable.^27^ Patients provided oral consent. The study was approved by the institutional review boards of the FHCRC and the University of The West Indies School of Clinical Medicine and Research.

## Results

### Model Calibration and Validation

Participants in the screening cohort consisted of 4300 men (median age, 54 years; range, 13-101 years) attending screening in Nassau from 2004 to 2018 and in Freeport from 2013 to 2018. A combined 8720 screening tests were performed for the men (2466 men in Nassau and 1834 in Freeport), representing approximately 5% of men aged 40 to 70 years.

The models modestly underprojected incidence without screening for men aged 55 to 69 years and modestly overprojected it for those aged 70 to 84 years (eFigure 1 in the Supplement). The models also modestly underprojected the prostate cancer mortality rates for men aged 55 to 69 years, whereas the projected mortality rates in the other age groups were reasonably close to the observed rates.

When the calibrated models were used to approximate the local screening program, the results for the proportion of men with PSA level higher than 10 ng/mL were within the range of results between Nassau and Freeport or slightly below the observed proportion (eFigure 2 in the Supplement). The models also reasonably reproduced the proportion of cancers with Gleason scores lower than or equal to 7 at diagnosis (eFigure 3 in the Supplement). A low proportion was projected by the FHCRC model but not the MISCAN model for men aged 55 years or younger (see the Discussion).

### Projected Prostate Cancer Incidence and Mortality

**Figure 1.**
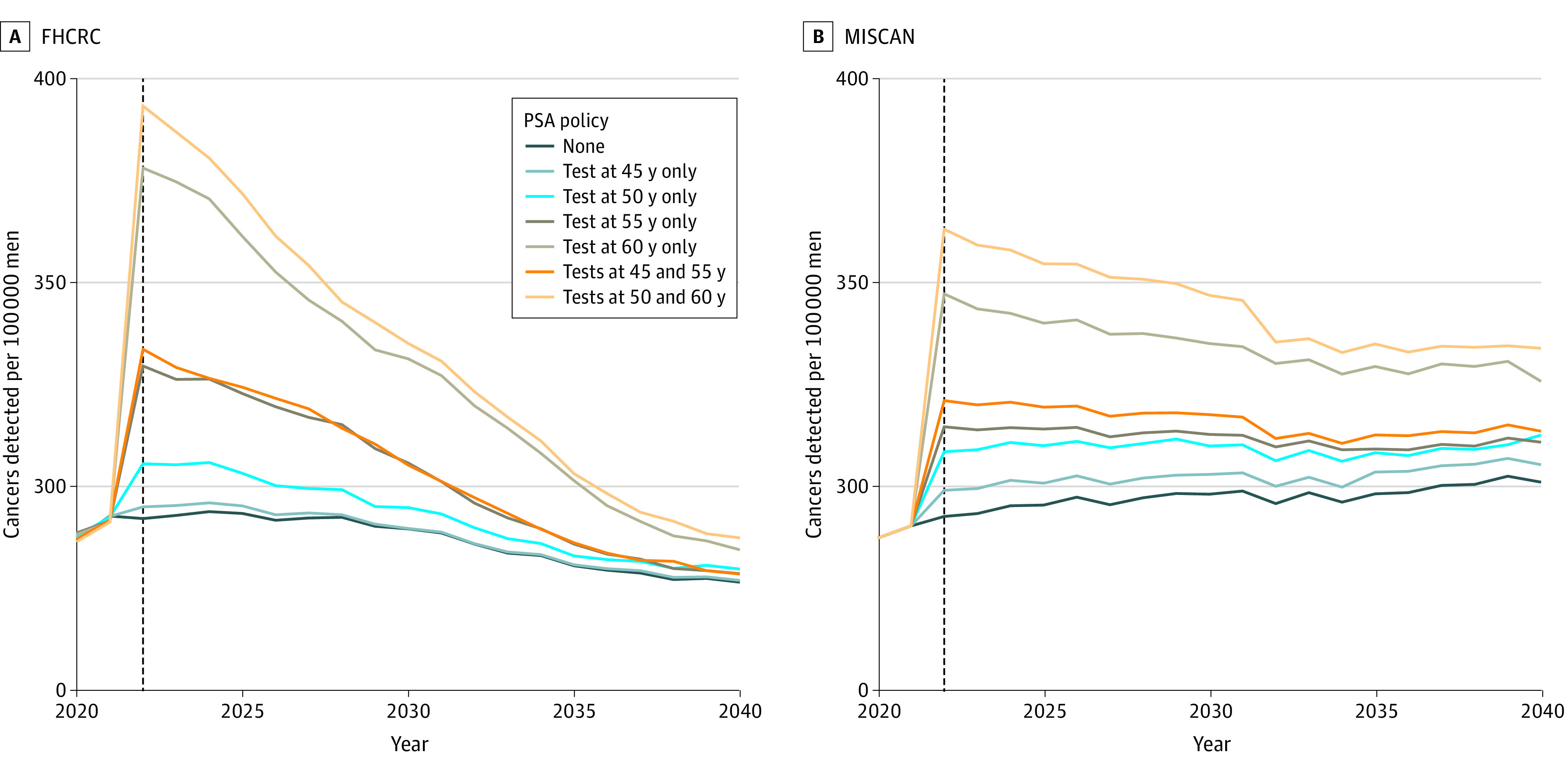
Projected Age-Adjusted Prostate Cancer Incidence Rates Under No Screening and Specified Screening Strategies Incidence rates projected by the 2 models have slightly increasing or decreasing secular trends owing to different methods for modeling population counts. FHCRC indicates Fred Hutchinson Cancer Research Center; MISCAN, Erasmus Medical Center Microsimulation Screening Analysis; and PSA, prostate-specific antigen.

Without screening, the 2 models projected approximately constant prostate cancer incidence trends (Figure 1). Under screening, both models projected sharp increases in incidence when the screening programs started, with the highest increase for the strategy with tests at 50 and 60 years of age. Thereafter, the models projected a decrease in incidence, which was less pronounced under the MISCAN model.

**Figure 2.**
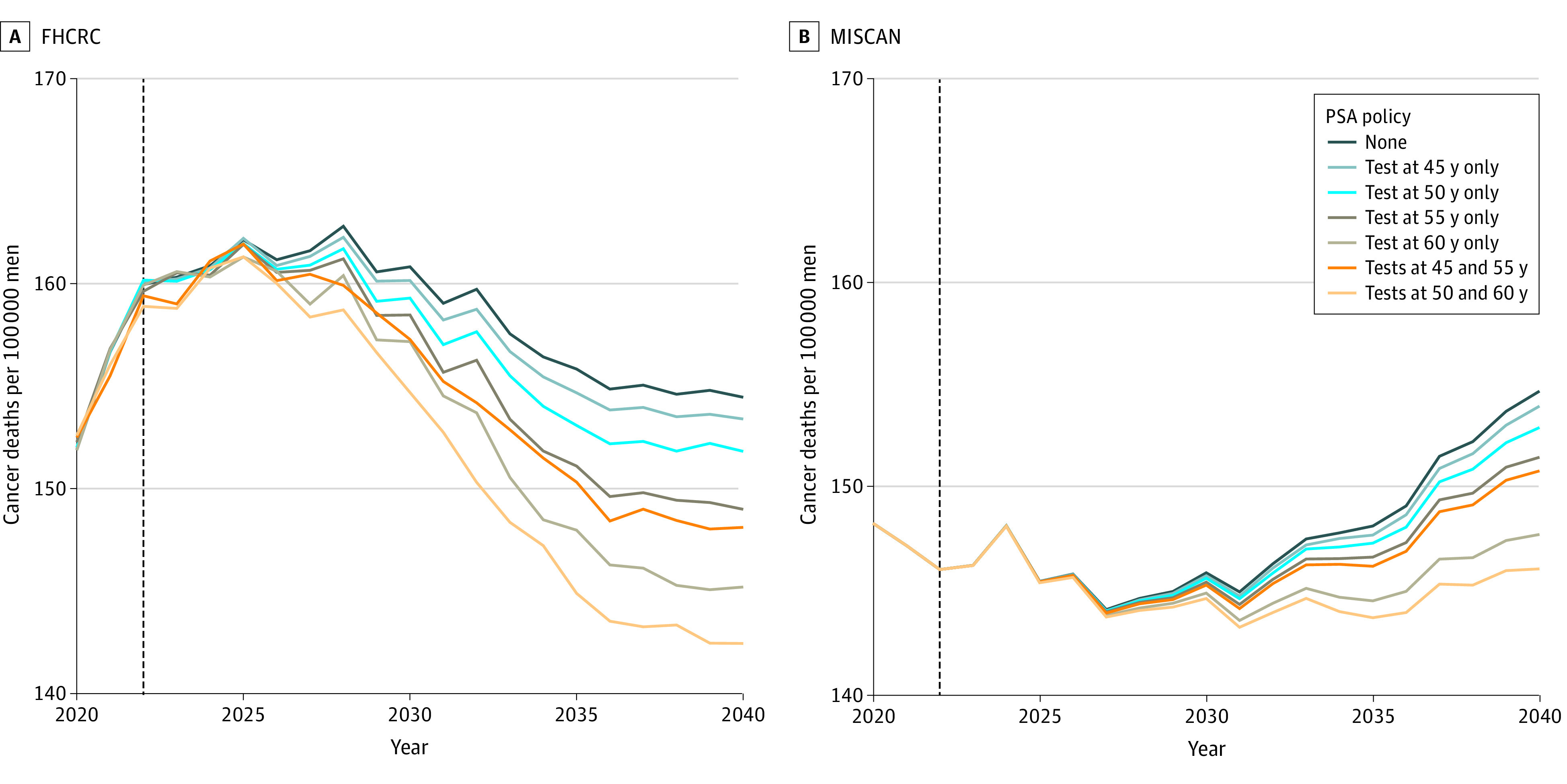
Projected Age-Adjusted Prostate Cancer Mortality Rates Under No Screening and Specified Screening Strategies Mortality rates projected by the 2 models have slightly increasing or decreasing secular trends owing to different methods for modeling population counts. FHCRC indicates Fred Hutchinson Cancer Research Center; MISCAN, Erasmus Medical Center Microsimulation Screening Analysis; and PSA, prostate-specific antigen.

Projected prostate cancer mortality rates without screening were approximately constant over time, with 154 to 155 deaths per 100 000 men aged 40 to 84 years in 2040 (Figure 2). The models projected decreasing mortality rates under screening, with the most pronounced decrease for the strategy with tests at 50 and 60 years of age (143-146 deaths per 100 000 men aged 40-84 years in 2040).

### Projected Absolute Outcomes and Harm-Benefit Trade-offs

**Figure 3.**
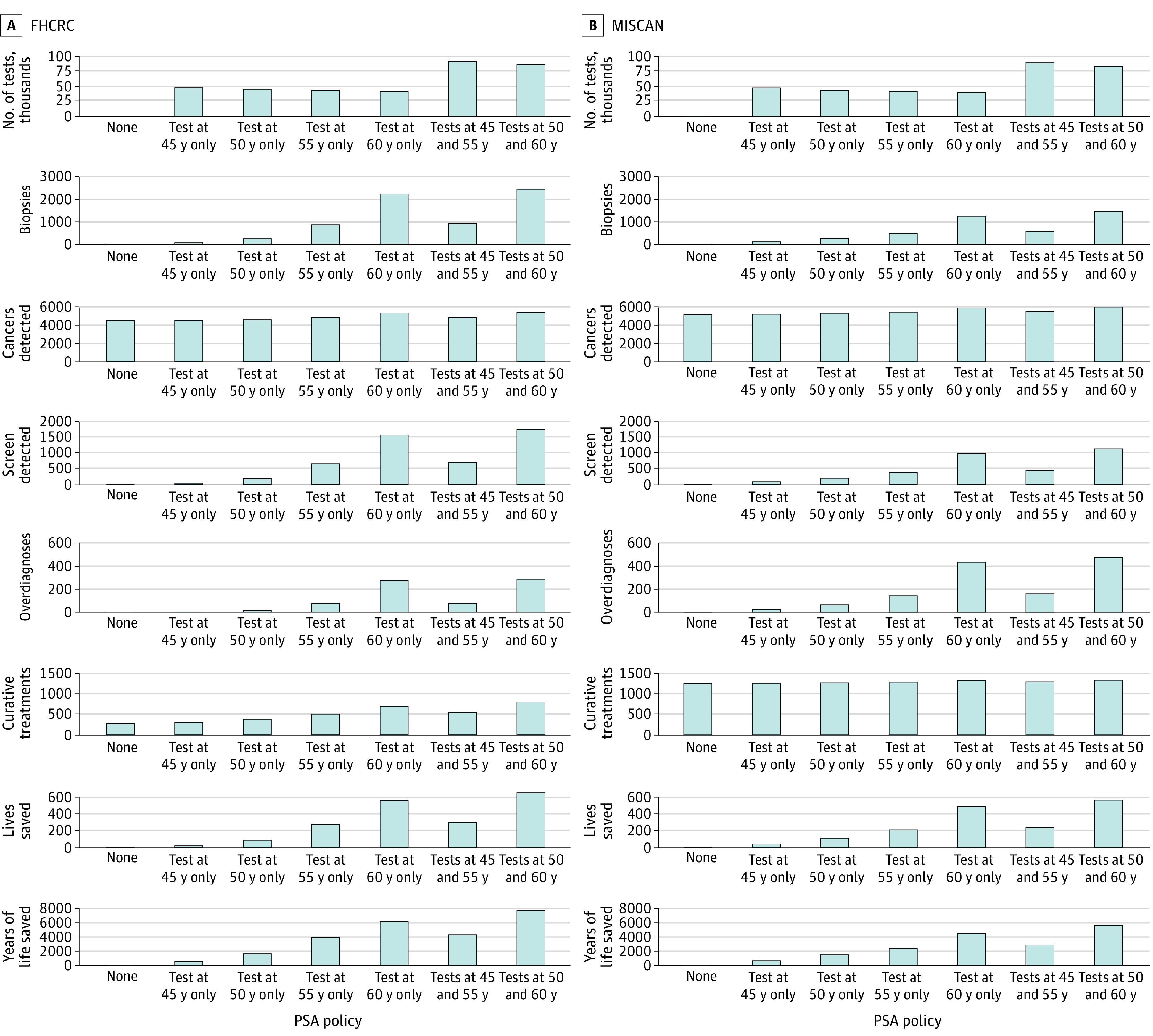
Projected Absolute Numbers of Medical Resources and Corresponding Mortality Benefits for Specified Screening Strategies Absolute numbers of prostate cancer deaths without screening projected by the 2 models were 9610 (FHCRC) and 13 728 (MISCAN). FHCRC indicates Fred Hutchinson Cancer Research Center; MISCAN, Erasmus Medical Center Microsimulation Screening Analysis; and PSA, prostate-specific antigen.

Absolute numbers of events projected for the Bahamian population from 2022 to 2040 are presented in Figure 3. Without screening, the models projected between 9610 and 13 686 prostate cancer deaths under lifetime follow-up of men aged 40 to 84 years during this period. Screening once at 50 years of age resulted in 87 to 111 lives saved, whereas screening once at 60 years of age resulted in 481 to 554 lives saved. Screening at both 50 and 60 years of age only slightly improved lives saved (15% to 16%) over onetime screening but doubled the number of tests and led to more biopsies and overdiagnoses. (Conclusions based on life-years gained instead of lives saved were similar.) Although the absolute numbers differed between models, especially for projected numbers of curative treatments owing to differing stage and grade distributions (eFigure 4 in the Supplement), the models projected similar patterns of resource requirements across screening strategies.

We also compared screening strategies by dividing the resources required by the number of lives saved (Table). Among onetime strategies, screening once at 60 years of age resulted in the fewest tests (range between models, 74-84 tests) and curative treatments (1.2-2.8 treatments) per life saved. In contrast, 1 screening at 50 years of age resulted in the fewest biopsies (2.3-2.5 vs 2.6-3.8 biopsies) and overdiagnoses (0.1-0.6 vs 0.5-0.9 overdiagnoses) per life saved.

**Table.** Numbers of Tests, Biopsies, Overdiagnoses, and Curative Treatments per Life Saved for Screening Strategies With Selected Testing Ages Projected by the MISCAN and FHCRC Models

Age, y	Tests/life saved		Biopsies/life saved		Overdiagnoses/life saved		Treatments/life saved	
	MISCAN model	FHCRC model	MISCAN model	FHCRC model	MISCAN model	FHCRC model	MISCAN model	FHCRC model
45	1144	2305	2.6	2.7	0.6	0.1	29.8	14.6
50	395	518	2.3	2.5	0.6	0.1	11.4	4.4
55	202	157	2.3	3.0	0.7	0.3	6.2	1.8
60	84	74	2.6	3.8	0.9	0.5	2.8	1.2
45 And 55	381	301	2.4	2.8	0.7	0.2	5.5	1.8
50 And 60	151	134	2.6	3.7	0.9	0.4	2.4	1.2

Abbreviations: FHCRC, Fred Hutchinson Cancer Research Center; MISCAN, Medical Center Microsimulation Screening Analysis.

### Sensitivity Analysis

Using a stage shift instead of cure-rate benefit of screening increased lives saved in one model (MISCAN) and decreased lives saved in the other (FHCRC) (eFigure 5 in the Supplement). Although absolute projections were sensitive to the mechanism of benefit, the relative benefits and harm-benefit trade-offs across strategies were highly consistent (eTable 1 and eFigure 5 in the Supplement).

## Discussion

Prostate cancer screening in a high-risk population is potentially highly beneficial. However, it is unclear whether it can be implemented in a manner that is sustainable and beneficial in a low-resource setting. In this study, we explored implications of limited screening strategies in the Bahamas, which represents a low-resource, high-risk setting. For example, a strategy of onetime screening at 60 years of age with perfect adherence is expected to involve 40 000 to 42 000 tests, 1200 to 2200 biopsies, 700 to 800 additional diagnoses, and 100 to 400 additional curative treatments during the next 18 years (Figure 3). This strategy is expected to prevent 500 to 600 of 10 000 to 14 000 prostate cancer deaths.

We studied the Bahamas as an example of a low-resource, high-risk setting because the data from the pilot screening program provided critical targets for calibrating and validating decision analytical models of screening strategies. In practice, however, these data are not population based, and they may not be representative of the population. Indeed, data from Nassau and Freeport yielded somewhat inconsistent results, underscoring this reality. The models do not incorporate any selection factors into who presents for screening (eg, men with early symptoms or family history), but we cannot rule out that men presenting for screening could have been a selective subset of the population. Despite the differences between modeled and observed screening outcomes, our models agree qualitatively about the absolute magnitude of the resources and outcomes of the screening programs considered.

Given that the models project only modest reductions in prostate cancer mortality across the strategies considered, it is important to assess whether such programs are worthwhile by considering resource demands and harm-benefit trade-offs. The harm-benefit trade-offs of screening programs were also broadly consistent between the 2 models and were favorable relative to estimates for more intensive programs in high-income countries. For example, for a onetime test at 60 years of age, the models projected 74 to 84 tests, 2.6 to 3.8 biopsies, and less than 1 overdiagnosed case per life saved. The MISCAN model previously found that for annual screening of mostly White men aged 55 to 69 years, 916 tests and 32 biopsies were needed to save 1 life, with 5 overdiagnosed cases.^28^ Another model, also based on screening as performed in the ERSPC, projected 385 men needed to be screened to save 1 life, with 11 overdiagnosed cases.^29^

Yet capacity constraints in the Bahamas imply that even strategies with favorable harm-benefit trade-offs may not be feasible. Currently, few urologists are employed in either the government system or the private sector.^30^ Many patients with adequate means seek primary care elsewhere, most often in Florida, which is less than an hour’s flight away. Among men with nonmetastatic disease treated in the Bahamas, most undergo radiation treatment at a single center.^31^ Our results support investment in additional surgical and radiation oncology services and provide estimates that can be used to determine the requisite clinicians and equipment. Additional research would be of value to quantify local resources that could be marshaled for an organized screening program.^32^

### Limitations

Few men of African descent were included in pivotal trials of PSA screening and definitive treatment. Although the 2 models each considered 2 mechanisms by which early detection and treatment could reduce mortality, they assumed that efficacy in men of African descent is similar to that reported by studies of predominantly White men. In addition, prostate cancer incidence and mortality rates were available for only a single year and could have been underestimated.

Although the models reasonably reproduced incidence rates in the Bahamas by age and proportions of men with PSA levels higher than 10 ng/mL in the screening program, there were moderate differences in the projected proportion of cancer cases with Gleason scores lower than or equal to 7. In the MISCAN model, men can progress from a lower to a higher Gleason score, and therefore older men tend to have higher Gleason scores. In the FHCRC model, older men also tend to have higher Gleason scores (which are fixed at onset) when unselected, but men with higher Gleason scores also have higher PSA growth rates, so those with PSA levels higher than 10 ng/mL at a young age tend to have high Gleason scores.

Differences between the models’ structures also explain differences in projected incidence trends. The MISCAN model projects higher rates of overdiagnosis than the FHCRC model, which implies that increases in incidence owing to screening at the start of the program are determined less by early detections that correspond to offsetting decreases in later years. Because overdiagnosis is unobservable, this outcome cannot be directly verified.

Finally, because treatments given in the Bahamas are likely to be different from those in the ERSPC, the cure-rate screening benefit calibrated to the ERSPC may not be appropriate. Replacing the cure rate with the stage shift resulted in different effects in the 2 models, but this is not unexpected because the stage-shift implementation depends on lead time, which differed between the models.^26,33^ Despite these differences between the models, both absolute and relative outcomes were qualitatively in agreement, providing a degree of confidence that they can help guide screening policy.

## Conclusions

In this decision analytical modeling study of prostate cancer screening programs in the Bahamas, we found that limited screening programs could provide modest benefit and quantify the resources that will be required for their sustainable implementation. Our use of 2 independently developed decision models illustrates uncertainty in the quantitative results because of different model structures and strengthens the credibility of the qualitative results. We consider this study a prototype for modeling other low-resource settings when local data are available for tailored model calibration.

## Notes

Supplement. **eFigure 1.** Prostate Cancer Incidence and Mortality per 100,000 Men (Crude Rate) in the Bahamas in 2018 Reported in GLOBOCAN Data and Projected by Erasmus Medical Center Microsimulation Screening Analysis (MISCAN) and the Fred Hutchinson Cancer Research Center (FHCRC) Models **eFigure 2.** Percentage of Men With PSA Greater Than 10 ng/mL Reported in a Local Screening Program in Freeport and Nassau and Projected by Erasmus Medical Center Microsimulation Screening Analysis (MISCAN) and the Fred Hutchinson Cancer Research Center (FHCRC) Models **eFigure 3.** Percentage of Cancers With Gleason Score Less Than or Equal to 7 Reported in a Local Screening Program in Freeport and Nassau and Projected by Erasmus Medical Center Microsimulation Screening Analysis (MISCAN) and the Fred Hutchinson Cancer Research Center (FHCRC) Models **eFigure 4.** Percentage of Cancers With Selected Combinations of Grade and Stage Under No Screening and Screening Strategies With Selected Testing Ages Projected by Erasmus Medical Center Microsimulation Screening Analysis (MISCAN) and the Fred Hutchinson Cancer Research Center (FHCRC) Models **eFigure 5.** Absolute Numbers of Lives Saved and Years of Life Saved Under Lifetime Follow-up Projected by the Erasmus Medical Center Microsimulation Screening Analysis (MISCAN) and the Fred Hutchinson Cancer Research Center (FHCRC) Models for Screening Strategies With Selected Testing Ages for the Bahamas for 2022-2040 Assuming a Stage-Shift Benefit **eFigure 6.** Fitted (Colored Lines) vs Empirical Kaplan-Meier (Black Lines) Estimates of the Cumulative Incidence of Prostate Cancer Death in the Black SEER 1980-1986 Population With a Diagnosis of Local/Regional Prostate Cancer Who Received Conservative Management **eFigure 7.** Fitted (Blue Lines) vs Empirical Kaplan-Meier (Black Lines) Estimates of the Cumulative Incidence of Prostate Cancer Death in the SEER 1980-1986 Population With a Diagnosis of Distant-Stage Prostate Cancer **eTable 1.** Number of Tests, Biopsies, Overdiagnoses, and Curative Treatments per Life Saved for Screening Strategies With Selected Testing Ages Projected by the Erasmus Medical Center Microsimulation Screening Analysis (MISCAN) and the Fred Hutchinson Cancer Research Center (FHCRC) Models Assuming a Stage-Shift Benefit Associated With Screening **eTable 2.** Cox Regression Results for Local/Regional-Stage Prostate Cancer **eTable 3.** Cox Regression Results for Distant-Stage Prostate Cancer **eMethods.** Baseline Prostate Cancer Survival Models
