# Supplementary material for: Evaluation of Prostate Cancer Screening Strategies in a Low-Resource, High-risk Population in the Bahamas
Source: JAMA Health Forum. 2022 May 20;3(5):e221116. doi: 10.1001/jamahealthforum.2022.1116 (PMC9123504; doi:10.1001/jamahealthforum.2022.1116)
Supplement: Supplement. — eFigure 1. Prostate Cancer Incidence and Mortality per 100,000 Men (Crude Rate) in the Bahamas in 2018 Reported in GLOBOCAN Data and Projected by Erasmus Medical Center Microsimulation Screening Analysis (MISCAN) and the Fred Hutchinson Cancer Research Center (FHCRC) Models eFigure 2. Percentage of Men With PSA Greater Than 10 ng/mL Reported in a Local Screening Program in Freeport and Nassau and Projected by Erasmus Medical Center Microsimulation Screening Analysis (MISCAN) and the Fred Hutchinson Cancer Research Center (FHCRC) Models eFigure 3. Percentage of Cancers With Gleason Score Less Than or Equal to 7 Reported in a Local Screening Program in Freeport and Nassau and Projected by Erasmus Medical Center Microsimulation Screening Analysis (MISCAN) and the Fred Hutchinson Cancer Research Center (FHCRC) Models eFigure 4. Percentage of Cancers With Selected Combinations of Grade and Stage Under No Screening and Screening Strategies With Selected Testing Ages Projected by Erasmus Medical Center Microsimulation Screening Analysis (MISCAN) and the Fred Hutchinson Cancer Research Center (FHCRC) Models eFigure 5. Absolute Numbers of Lives Saved and Years of Life Saved Under Lifetime Follow-up Projected by the Erasmus Medical Center Microsimulation Screening Analysis (MISCAN) and the Fred Hutchinson Cancer Research Center (FHCRC) Models for Screening Strategies With Selected Testing Ages for the Bahamas for 2022-2040 Assuming a Stage-Shift Benefit eFigure 6. Fitted (Colored Lines) vs Empirical Kaplan-Meier (Black Lines) Estimates of the Cumulative Incidence of Prostate Cancer Death in the Black SEER 1980-1986 Population With a Diagnosis of Local/Regional Prostate Cancer Who Received Conservative Management eFigure 7. Fitted (Blue Lines) vs Empirical Kaplan-Meier (Black Lines) Estimates of the Cumulative Incidence of Prostate Cancer Death in the SEER 1980-1986 Population With a Diagnosis of Distant-Stage Prostate Cancer eTable 1. Number of Tests, Biopsies, Overdiagnoses, [file jamahealthforum-e221116-s001.pdf]

Supplemental Online Content

Heijnsdijk EAM, Gulati R, Lange JM, Tsodikov A, Roberts R, Etzioni R. Evaluation of prostate cancer screening strategies in a low-resource, high-risk population in the Bahamas. *JAMA Health Forum*. 2022;3(5):e221116. doi:10.1001/jamahealthforum.2022.1116

eFigure 1. Prostate Cancer Incidence and Mortality per 100,000 Men (Crude Rate) in the Bahamas in 2018 Reported in GLOBOCAN Data and Projected by Erasmus Medical Center Microsimulation Screening Analysis (MISCAN) and the Fred Hutchinson Cancer Research Center (FHCRC) Models

eFigure 2. Percentage of Men With PSA Greater Than 10 ng/mL Reported in a Local Screening Program in Freeport and Nassau and Projected by Erasmus Medical Center Microsimulation Screening Analysis (MISCAN) and the Fred Hutchinson Cancer Research Center (FHCRC) Models

eFigure 3. Percentage of Cancers With Gleason Score Less Than or Equal to 7 Reported in a Local Screening Program in Freeport and Nassau and Projected by Erasmus Medical Center Microsimulation Screening Analysis (MISCAN) and the Fred Hutchinson Cancer Research Center (FHCRC) Models

eFigure 4. Percentage of Cancers With Selected Combinations of Grade and Stage Under No Screening and Screening Strategies With Selected Testing Ages Projected by Erasmus Medical Center Microsimulation Screening Analysis (MISCAN) and the Fred Hutchinson Cancer Research Center (FHCRC) Models

eFigure 5. Absolute Numbers of Lives Saved and Years of Life Saved Under Lifetime Follow-up Projected by the Erasmus Medical Center Microsimulation Screening Analysis (MISCAN) and the Fred Hutchinson Cancer Research Center (FHCRC) Models for Screening Strategies With Selected Testing Ages for the Bahamas for 2022-2040 Assuming a Stage-Shift Benefit

eTable 1. Number of Tests, Biopsies, Overdiagnoses, and Curative Treatments per Life Saved for Screening Strategies With Selected Testing Ages Projected by the Erasmus Medical Center Microsimulation Screening Analysis (MISCAN) and the Fred Hutchinson Cancer Research Center (FHCRC) Models Assuming a Stage-Shift Benefit Associated With Screening

eMethods. Baseline Prostate Cancer Survival Models

eTable 2. Cox Regression Results for Local/Regional-Stage Prostate Cancer

eTable 3. Cox Regression Results for Distant-Stage Prostate Cancer

eFigure 6. Fitted (Colored Lines) vs Empirical Kaplan-Meier (Black Lines) Estimates of the Cumulative Incidence of Prostate Cancer Death in the Black SEER 1980-1986 Population With a Diagnosis of Local/Regional Prostate Cancer Who Received Conservative Management

© 2022 Heijnsdijk et al. *JAMA Health Forum.*

eFigure 7. Fitted (Blue Lines) vs Empirical Kaplan-Meier (Black Lines) Estimates of the Cumulative Incidence of Prostate Cancer Death in the SEER 1980-1986 Population With a Diagnosis of Distant-Stage Prostate Cancer

This supplemental material has been provided by the authors to give readers additional information about their work.

© 2022 Heijnsdijk et al. *JAMA Health Forum.*

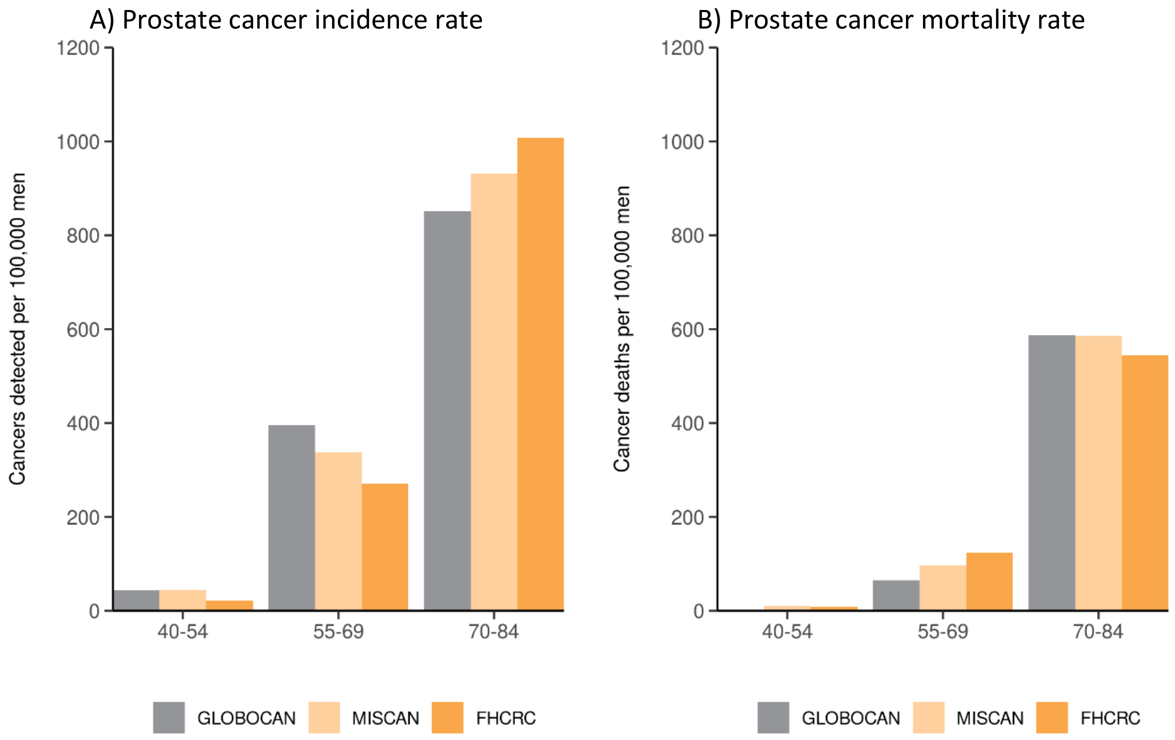

eFigure 1. Prostate Cancer Incidence and Mortality per 100,000 Men (Crude Rate) in the Bahamas in 2018 Reported in GLOBOCAN Data and Projected by Erasmus Medical Center Microsimulation Screening Analysis (MISCAN) and the Fred Hutchinson Cancer Research Center (FHCRC) Models

This supplemental material has been provided by the authors to give readers additional information about their work.

© 2022 Heijnsdijk et al. *JAMA Health Forum.*

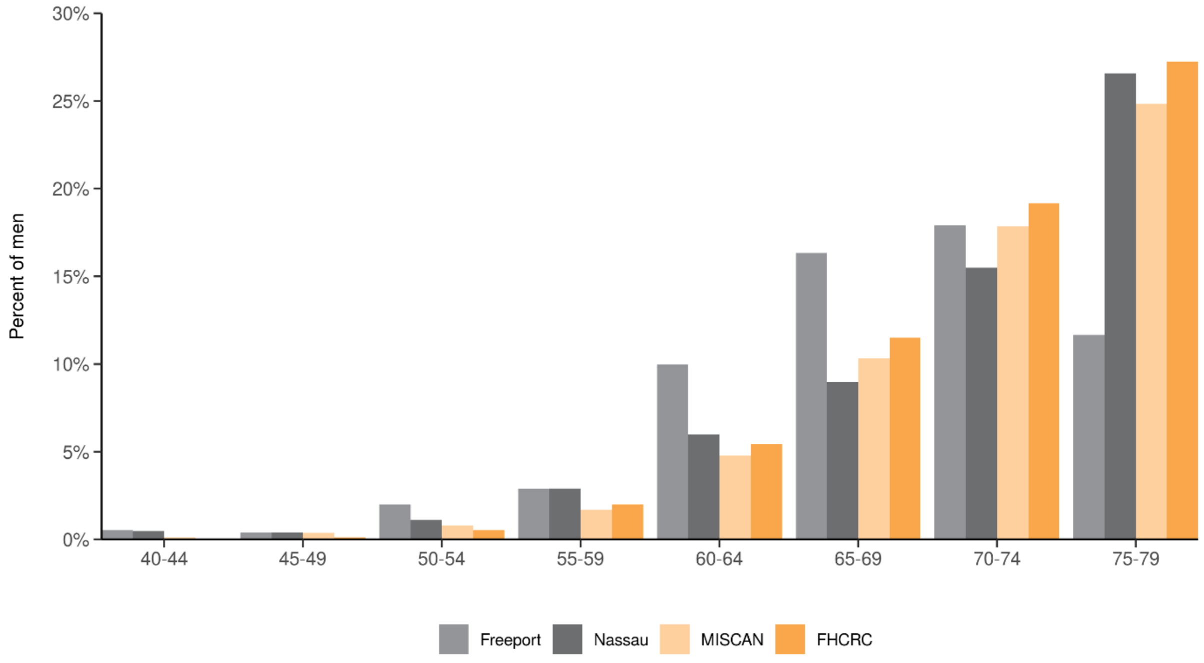

eFigure 2. Percentage of Men With PSA Greater Than 10 ng/mL Reported in a Local Screening Program in Freeport and Nassau and Projected by Erasmus Medical Center Microsimulation Screening Analysis (MISCAN) and the Fred Hutchinson Cancer Research Center (FHCRC) Models

This supplemental material has been provided by the authors to give readers additional information about their work.

© 2022 Heijnsdijk et al. *JAMA Health Forum.*

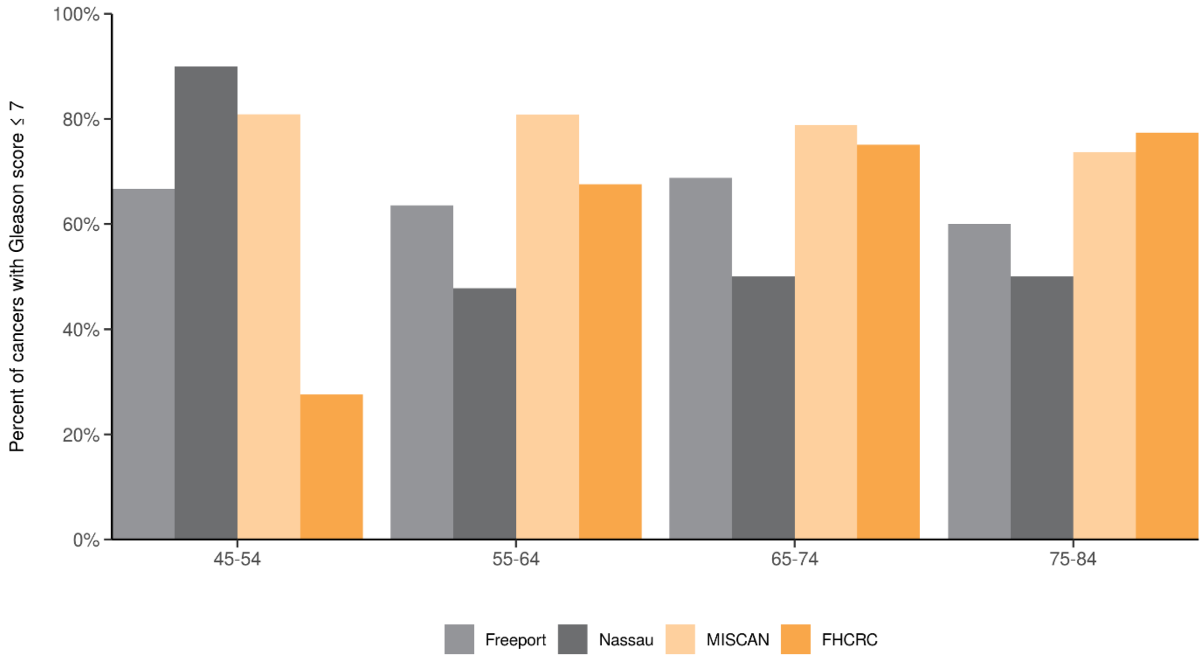

eFigure 3. Percentage of Cancers With Gleason Score Less Than or Equal to 7 Reported in a Local Screening Program in Freeport and Nassau and Projected by Erasmus Medical Center Microsimulation Screening Analysis (MISCAN) and the Fred Hutchinson Cancer Research Center (FHCRC) Models

This supplemental material has been provided by the authors to give readers additional information about their work.

© 2022 Heijnsdijk et al. *JAMA Health Forum.*

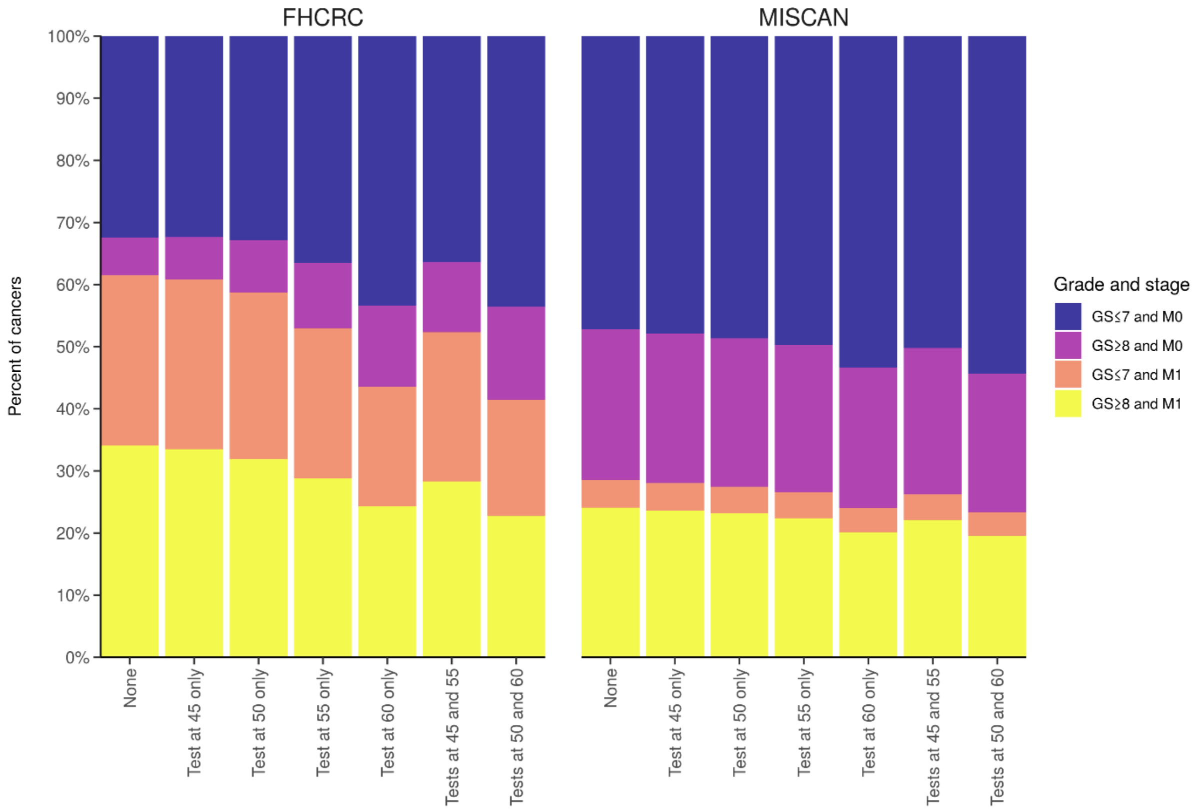

eFigure 4. Percentage of Cancers With Selected Combinations of Grade and Stage Under No Screening and Screening Strategies With Selected Testing Ages Projected by Erasmus Medical Center Microsimulation Screening Analysis (MISCAN) and the Fred Hutchinson Cancer Research Center (FHCRC) Models

This supplemental material has been provided by the authors to give readers additional information about their work.

© 2022 Heijnsdijk et al. *JAMA Health Forum.*

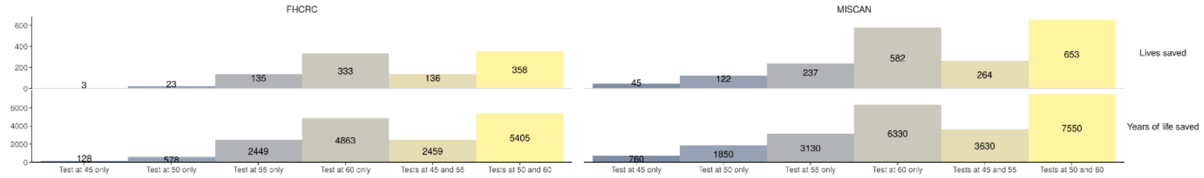

eFigure 5. Absolute Numbers of Lives Saved and Years of Life Saved Under Lifetime Follow-up Projected by the Erasmus Medical Center Microsimulation Screening Analysis (MISCAN) and the Fred Hutchinson Cancer Research Center (FHCRC) Models for Screening Strategies With Selected Testing Ages for the Bahamas for the Period 2022-2040 Assuming a Stage-Shift Benefit

Absolute numbers of prostate cancer deaths without screening projected by the two models were 9,610 (FHCRC) and 13,728 (MISCAN).

This supplemental material has been provided by the authors to give readers additional information about their work.

© 2022 Heijnsdijk et al. *JAMA Health Forum.*

eTable 1. Number of Tests, Biopsies, Overdiagnoses, and Curative Treatments per Life Saved for Screening Strategies With Selected Testing Ages Projected by the Erasmus Medical Center Microsimulation Screening Analysis (MISCAN) and the Fred Hutchinson Cancer Research Center (FHCRC) Models Assuming a Stage-Shift Benefit Associated With Screening

Testing ages in years	Tests/life saved		Biopsies/life saved		Overdiagnoses/life saved		Treatments/life saved	
	MISCAN	FHCRC	MISCAN	FHCRC	MISCAN	FHCRC	MISCAN	FHCRC
45 only	1,070	24,124	2.4	28.5	0.5	1.0	28.0	153
50 only	360	2,071	2.1	10.1	0.5	0.6	10.4	17.5
55 only	177	320	2.0	6.1	0.6	0.5	5.4	3.7
60 only	69	123	2.1	6.4	0.8	0.8	2.3	2.1
45 and 55	342	627	2.1	5.8	0.6	0.5	4.9	3.7
50 and 60	129	238	2.2	6.5	0.7	0.8	2.1	2.2

This supplemental material has been provided by the authors to give readers additional information about their work.

© 2022 Heijnsdijk et al. *JAMA Health Forum.*

eMethods. Baseline Prostate Cancer Survival Models

We fit Cox regression models to SEER cancer-specific survival data for Black men diagnosed with local/regional or distant stage prostate cancer in the pre-screening era who did not receive curative treatment. These baseline prostate cancer survival models were modified to reflect the effects of screening and curative treatment as described in the main text. Here we describe the baseline survival models.

Men were eligible for inclusion if they were diagnosed with local/regional or distant stage prostate cancer during the period 1980-1986 and were aged 50-84 years at diagnosis. In SEER, there were 3,291 men diagnosed with local/regional and 1,260 men diagnosed with distant stage disease.

Primary treatment variables were based on SEER variables "Therapy.Radiation" and "Therapy.Site specific surgery (1983-1997)." The "Other/none" category includes men who either did not receive radical prostatectomy or radiation or for whom surgery status is unknown. Given the historical treatment patterns at the time, the "Other/none" category encompasses men who received conservative management.

Covariates in the Cox regression model for local/regional stage prostate cancer included age at diagnosis and age-squared, centered at 70; historic grade (well/moderately differentiated or poorly/undifferentiated or unknown); primary treatment (radical prostatectomy, radiotherapy, or other/none); and three-way interaction terms between age, grade, and treatment and between age-squared, grade, and treatment.

Covariates in the Cox regression model for distant stage prostate cancer included grade, treatment, and interactions between grade and treatment. Treatment was categorized according to the same categories as for the local/regional model.

Supplementary Tables 2 and 3 show the results for the local/regional and distant stage models. Supplementary Figures 6 and 7 show corresponding empirical estimates of the cumulative probability of prostate cancer death and the fitted models.

After fitting the models, we obtained projections of survival with respect to years since diagnosis to population subgroups based on applying covariate level-effects to the baseline survival function extracted from each model. All projections for the local/regional model assumed that the men received conservative management. Projections were calculated by age in years and grade (local/regional stage model) or by grade (distant stage model) and averaged across five-year intervals to match the inputs for the microsimulation models.

This supplemental material has been provided by the authors to give readers additional information about their work.

eTable 2. Cox Regression Results for Local/Regional-Stage Prostate Cancer

Covariate	Hazard ratio	95% CI	P-value
Age at diagnosis	1.031	[1.015,1.048]	<.001
Age-squared	1.001	[0.999,1.003]	0.486
Grade (ref=well differentiated)			
Poorly/undifferentiated	3.65	[2.846,4.683]	<.001
Unknown	1.28	[0.923,1.776]	0.14
Primary treatment (ref=Other/none)			
Radical Prostatectomy	0.705	[0.548,0.906]	0.006
Radiotherapy	0.858	[0.594,1.237]	0.411
Age x Grade= poorly/undifferentiated	0.977	[0.951,1.004]	0.091
Age x Grade= unknown	1.010	[0.977,1.044]	0.556
Age x Treatment=radical prostatectomy	1.040	[1.014,1.067]	0.002
Age x Treatment = radiotherapy	0.974	[0.919,1.033]	0.385
Grade=poorly/undifferentiated x Treatment=radical prostatectomy	0.995	[0.67,1.478]	0.981
Grade=unknown x Treatment=radical prostatectomy	1.234	[0.74,2.058]	0.420
Grade=poorly/undifferentiated x Treatment=Radiotherapy	0.731	[0.42,1.27]	0.266
Grade=unknown x Treatment=Radiotherapy	1.000	[0.425,2.35]	0.999
Grade=poorly/undifferentiated x Age-squared	1.002	[1.000,1.004]	0.154
Grade=unknown x Age-squared	0.999	[0.995,1.003]	0.547
Treatment=radical prostatectomy x Age-squared	1.002	[1.000,1.004]	0.186
Treatment=radiotherapy x Age-squared	1.002	[0.996,1.008]	0.534
Age x Grade=poorly/undifferentiated x Treatment=radical prostatectomy	0.972	[0.93,1.017]	0.217
Age x Grade=unknown x Treatment=radical prostatectomy	0.959	[0.911,1.009]	0.111
Age x Grade=poorly/undifferentiated x Treatment=Radiotherapy	1.036	[0.952,1.127]	0.412
Age x Grade=unknown x Treatment = Radiotherapy	0.970	[0.864,1.088]	0.603
Grade=poorly/undifferentiated x Treatment=radical prostatectomy x Age-squared	0.996	[0.992,1.000]	0.084
Grade=unknown x Treatment=radical prostatectomy x Age-squared	1.000	[0.994,1.006]	0.980
Grade=poorly/undifferentiated x Treatment=radiotherapy x Age-squared	1.001	[0.993,1.009]	0.817
Grade=Unknown x Treatment=radiotherapy x Age-squared	1.002	[0.992,1.012]	0.656

This supplemental material has been provided by the authors to give readers additional information about their work.

eTable 3. Cox Regression Results for Distant-Stage Prostate Cancer

Covariate	Hazard ratio	95% CI	P-value
Grade (ref=well differentiated)			
Poorly/undifferentiated	1.643	[1.375,1.965]	<.001
Unknown	1.445	[1.195,1.747]	<.001
Primary treatment (ref=Other/none)			
Radical Prostatectomy	0.749	[0.566,0.991]	0.043
Radiotherapy	0.315	[0.155,0.64]	0.001
Grade=poorly/undifferentiated x Treatment=radical prostatectomy	1.199	[0.833,1.727]	0.328
Grade=unknown/Treatment=radical prostatectomy	0.71	[0.449,1.124]	0.142
Grade=poorly/undifferentiated x Treatment=radiotherapy	4.824	[2.123,10.971]	<.001
Grade=unknown/Treatment=radiotherapy	2.373	[0.702,8.014]	0.164

This supplemental material has been provided by the authors to give readers additional information about their work.

© 2022 Heijnsdijk et al. *JAMA Health Forum.*

eFigure 6. Fitted (Colored Lines) vs Empirical Kaplan-Meier (Black Lines) Estimates of the Cumulative Incidence of Prostate Cancer Death in the Black SEER 1980-1986 Population With a Diagnosis of Local/Regional Prostate Cancer Who Received Conservative Management

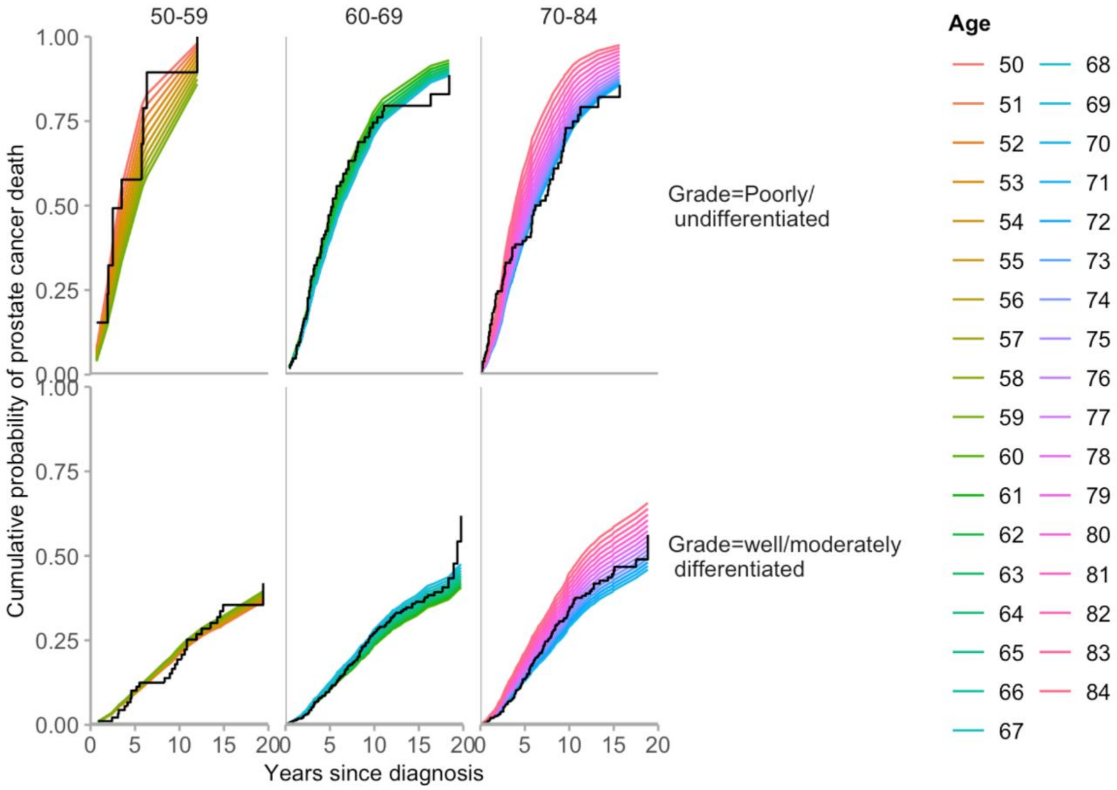

This supplemental material has been provided by the authors to give readers additional information about their work.

© 2022 Heijnsdijk et al. *JAMA Health Forum.*

eFigure 7. Fitted (Blue Lines) vs Empirical Kaplan-Meier (Black Lines) Estimates of the Cumulative Incidence of Prostate Cancer Death in the SEER 1980-1986 Population With a Diagnosis of Distant-Stage Prostate Cancer

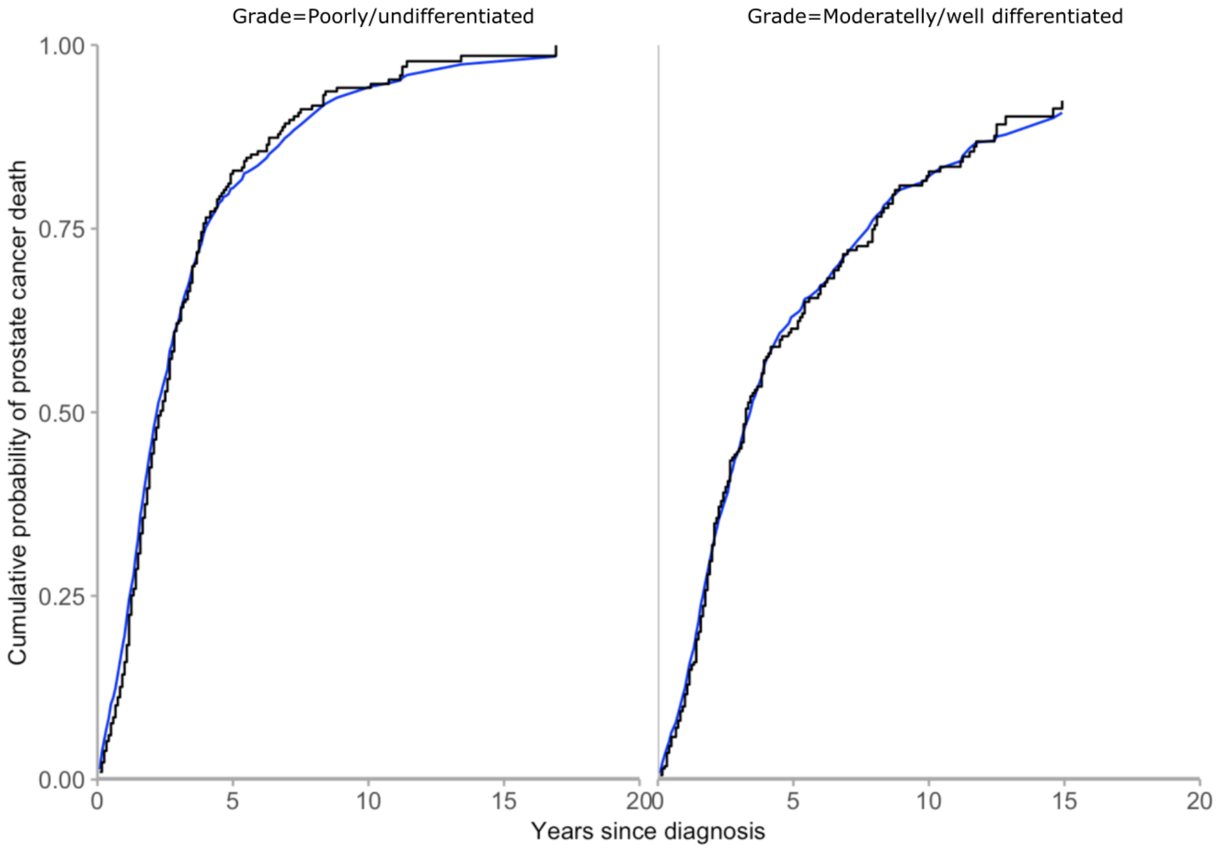

This supplemental material has been provided by the authors to give readers additional information about their work.

© 2022 Heijnsdijk et al. *JAMA Health Forum.*
